# Physical–Chemical Aspects of the Preparation and Drug Release of Electrospun Scaffolds

**DOI:** 10.3390/pharmaceutics13101645

**Published:** 2021-10-09

**Authors:** Lu Cui, Judit Rebeka Molnár, Mária Budai-Szűcs, Mária Szécsényi, Katalin Burián, Péter Vályi, Szilvia Berkó, Béla Pukánszky

**Affiliations:** 1 Laboratory of Plastics and Rubber Technology, Department of Physical Chemistry and Materials Science, Budapest University of Technology and Economics, H-1521 Budapest, Hungary; molnar.judit.rebeka@gmail.com (J.R.M.); pukanszky.bela@vbk.bme.hu (B.P.); 2 Institute of Materials and Environmental Chemistry, Research Centre for Natural Sciences, ELKH Eötvös Loránd Research Network, H-1519 Budapest, Hungary; 3 Institute of Pharmaceutical Technology and Regulatory Affairs, Faculty of Pharmacy, University of Szeged, H-6720 Szeged, Hungary; budai-szucs.maria@szte.hu (M.B.-S.); berko.szilvia@szte.hu (S.B.); 4 Institute of Clinical Microbiology, Faculty of Medicine, University of Szeged, H-6720 Szeged, Hungary; szecsenyi.maria@med.u-szeged.hu (M.S.); burian.katalin@med.u-szeged.hu (K.B.); 5 Department of Oral Diagnostics, Faculty of Dentistry, Semmelweis University, H-1085 Budapest, Hungary; valyi.peter@dent.semmelweis-univ.hu

**Keywords:** PLA, amoxicillin, composition, structure, drug release, antimicrobial activity

## Abstract

Fibers were spun from a mixture of dichloromethane (DCM) and dimethyl sulfoxide (DMSO) solution of poly(lactic acid)(PLA) containing various amounts of amoxicillin (Amox) as the active component. Composition changes during spinning, structure, solubility, and the location of the drug were considered during the evaluation of drug release and microbial activity. The results showed that the composition of the material changes during the preparation procedure. The solubility of the drug in the components and that of the components in each other is limited, which results in the formation of several phases and the precipitation of the drug. The technology used results in the partitioning of the drug; some is located inside, while the rest is among the fibers. The wetting of the fibers or disks by the water-based dissolution media is poor, the penetration of the liquid into and the diffusion of the active component out of the device takes considerable time. Drug release takes place in one, burst-like step, only Amox located among the fibers dissolve and diffuse into the surrounding medium. The slow second stage of release claimed in the literature is less probable because the size of the Amox molecule is considerably larger than the holes creating the free volume of the polymer. The prepared device has antimicrobial activity, inhibits the growth of the two bacterial strains studied. The time scale of activity is short and corresponds to that of the release experiments and the burst-like behavior of the device. The results clearly prove that physical–chemical factors play a determining role in the effect and efficiency of medical devices prepared from electrospun fibers containing an active component.

## 1. Introduction

Drug delivery is a crucial question of modern therapy. The strength, time, and site of action of delivery may influence the recovery of the patient significantly. A good example is periodontitis, an inflammatory disease resulting from the overgrowth of subgingival polymicrobial community in susceptible hosts affecting the tissues surrounding the teeth. This disease is the most prevalent reason for tooth loss among adults [1]. Using systemic antibiotics is usually adjunctive with the elimination of biofilm in the periodontal treatment [2]. Systemically administered antibiotics may not be present in sufficient concentration in the periodontal pocket; therefore, they are often ineffective while the common side effects of antimicrobial therapy could occur [3].

Consequently, it is not very surprising that numerous papers deal with local delivery systems containing antimicrobial drugs, which alone or in combination with other procedures may result in a more efficient treatment. The introduction of devices containing antibiotics onto the exact location where the delivery is required may result in efficient therapy with limited side effects and reduced risk of developing drug-resistant microbes. Numerous local drug delivery systems such as fibers, strips, films, injectable gels, micro- and nanoparticulate systems, vesicular systems, and in-situ forming implants have been developed for this purpose in the last decades [3,4,5,6,7,8,9].

The pharmaceutical industry shows increasing interest in the electrospinning technique due to its extraordinary advantages, such as large processing flexibility, controlled drug release kinetics, and topical/systemic therapies compared with traditional drug formulations [10,11,12,13,14]. The physical–chemical properties of the polymer, the drugs, and the solvents greatly influence interactions and drug release kinetics—the compatibility of the drug and the solvent, the drug and the polymer, and polymer/solvent interactions, both in the spinning solution and in the fibers, as well as the evaporation of the solvent—all of which impact the location of the drug, encapsulation, and the solid-state characteristics of the formulation [15,16]. 

Amoxicillin (Amox) is one of the most important antibiotics, is relatively cheap and has broad antimicrobial activity, bactericidal effect, and high therapeutic index. Consequently, quite a few studies focus on the preparation and drug release of electrospun fibers prepared from biopolymers and Amox, in order to use them as drug delivery systems. These studies include the optimization of the preparation process, the modification of properties, the control of drug release, and microbiological effects [17,18,19,20,21,22,23,24,25,26,27,28]. Valarezo et al. [20,21], for example, prepared fibers from poly(lactic acid) (PLA)/polycaprolactone (PCL) blends containing Amox and could control the rate of drug release by adjusting the ratio of the two polymers. Zhang et al. [22] modified poly(d,l-lactide-co-glycolide) acid (PLGA) with methoxy-poly(ethylene glycol)-amine to improve the compatibility of Amox with the polymer and achieved good antibacterial efficacy, cytocompatibility, and hemocompatibility. Tang et al. [23] developed PLGA/collagen nanofibers by coaxial spinning and they could achieve a loading capacity of Amox exceeding 90%. Occasionally a drug carrier component (hydroxyapatite [24,25], halloysite nanotubes [26,27]) is also incorporated into the fibers in order to improve mechanical properties or control drug delivery.

Although more and more reports are published on devices fabricated from electrospun fibers containing an active component, mostly antibiotics, surprisingly little attention is paid to the physical–chemical aspects of the preparation or the drug release process. The authors usually assume that the composition of the fibers is the same as that of the spinning solution and the fibers are homogeneous. Homogeneous drug distribution is not assumed, because the particles of the drug are often observed on the surface of the fibers. The phase separation of the polar drug and the presence of the particles usually leads to a burst-like delivery, which is said to be followed by a slow, controlled release. The papers on electrospun biopolymer devices containing Amox unanimously agree on this two-step process irrespective of differences in the experimental details [17,21,27]. The fibers are spun from different solvents (acetone, chloroform/methanol, dichloromethane (DCM) or tetrahydrofolate (THF)/dimethylformamide (DMF)), Amox content changes between 1 and 7 wt%, the delivery medium is water, phosphate-buffered saline (PBS) or standard buffer solution, and a wide variety of polymers are used as a matrix (PLA, PLGA, PCL). No wonder that the extent of drug delivery also changes in a wide range, between 10 and 100% [19,20,21,25,26,27,28]. The interaction of the components, mutual miscibility, the structure of the matrix, and the distribution or partitioning of the active component are mentioned occasionally but rarely investigated, although all must influence the time dependence, extent, and efficiency of drug delivery very much.

In view of the considerations above, we prepared electrospun fibers from PLA and Amox, fabricated compressed disks from them, and determined drug release as well as the microbiological activity of the device. We focused our attention mainly on issues and effects often ignored or neglected in earlier studies, such as changing composition during the fabrication process, the location of the active component, the role of wetting and penetration in drug delivery, and the time dependence of release. All these questions are considered and discussed, keeping in mind the use of the prepared device for local periodontal treatment.

## 2. Experimental

### 2.1. Materials

PLA granules (Ingeo 4032D, density of 1.24 g/cm^3^) were supplied by NatureWorks (Minnetonka, MN, USA). Amoxicillin trihydrate was obtained from Tokyo Chemical Industry (Tokyo, Japan) with >98% purity and 11–15% water content. DCM, dimethyl sulfoxide (DMSO) and sodium chloride were purchased from Molar Chemicals Kft. (Halásztelek, Hungary). Physiological saline solution (saline) was prepared from water containing 0.9 wt% sodium chloride. Distilled water was used for the preparation of water-based solutions in the drug release tests.

### 2.2. Sample Preparation

PLA fibers containing various amounts of Amox were prepared by electrospinning at ambient temperature, 15 kV voltage, 15 cm collector distance, and a feeding rate of 2 µL/s. The solvent used was the 80/20 vol% mixture of DCM and DMSO. The solution contained the polymer in 8.8 wt%, while the amount of the active component was 0.25, 0.50, 0.96, and 1.23 wt% calculated for the amount of PLA. The aluminum foils supporting the collected fibers were put into an air circulating oven (Venti-Line VL115, VWR International, LLC, Lutterworth, UK) for 2 days at 40 °C to remove DMSO. Electrospun fiber mats taken directly from the foils and round, compressed disks were used for characterization, as well as the analysis of release kinetics and microbial activity. The disks were obtained by compressing approximately 50 mg fibers under 3 MPa pressure for 60 s in a pellet die of 13 mm diameter (Specac Atlas Manual Hydraulic Press 25T and Specac 13 mm Pellet Press Die (Specac Ltd., Orpington, Kent, UK). 

### 2.3. Characterization

The structure of the fiber mats and disks was characterized by using a JEOL JSM 6380LA (JEOL Ltd., Tokyo, Japan) scanning electron microscope (SEM). Disks were fractured at liquid nitrogen temperature and then a thin gold layer was sputtered onto the fracture surface prior to the SEM study. The crystalline structure of the components was studied by differential scanning calorimetry (DSC) and X-ray diffraction measurements (XRD). DSC measurements were carried out on 3–5 mg fibers or samples taken from the disks. The measurements were made using a Perkin Elmer DSC IC apparatus (PerkinElmer, Budapest, Hungary); samples were heated from 30–200 °C at the heating rate of 10 °C/min under N_2_ purge. XRD patterns were recorded using a Philips PW 1830 diffractometer (UK). Measurements were carried out in the range of 2θ angles of 5–40°, with 0.04° increments and 1s/step rate at the accelerating voltage of 40 kV and exciting current of 35 mA.

Solubility was determined by preparing over-saturated solutions of Amox in the corresponding solvent (DMSO, DCM, water) or solvent mixture (DCM/DMSO/PLA). The amount of dissolved Amox was determined by UV-Vis spectroscopy using a Unicam UV 500 type spectrophotometer (Unicam, Cambridge, UK) after calibration. The measurement was done in the wavelength range of 200–400 nm with a scan speed of 120 mm/min and lamp change at 320 nm. The concentration of the drug was determined from the intensity of the absorbance peak characteristic for Amox. The solubility of Amox in PLA was determined by the preparation of solvent-cast PLA/Amox films and subsequent UV-Vis spectroscopy. The characteristic absorbance of Amox increases linearly with concentration up to the solubility limit and then levels off as the drug forms a separate phase. The solubility parameter of the materials used in this study was determined by the group contribution approach using the values proposed by Fedors [29].

In order to determine the amount of encapsulated Amox, disks were immersed into 20 mL water and kept there for 5 days. After 5 days, the disks were taken out and washed with distilled water 3 times to remove the eluates, and then dried in an oven at 110 °C for 24 h. Subsequently, 4 mL DCM was added to dissolve the dried disks and then Amox was extracted with water by shaking the mixture thoroughly. The mixture was left standing overnight until the DCM/PLA and water/Amox phases separated. The amount of Amox was determined by UV-Vis spectrophotometry as described above.

The wetting of compressed PLA/Amox disks in contact with water was studied by using an Optical Contact Angle System (Ramé Hart 100-00 Goniometer, ramé- hart instrument co., Morris County, NJ, USA). The measurement was done by placing a water droplet of 10 μL volume from a calibrated syringe onto the surface. The contact angle was measured at 0, 10, 20, 30, and 40 min. Five parallel measurements were done on each sample. In order to determine the penetration of water, as well as the ultimate water uptake of the compressed disks, approximately 100 mg of material cut from the disk was immersed into 25 mL water for 104 h. The weight of the samples was recorded as a function of time and the degree of penetration was calculated.

The structure of the Amox molecule was modeled using the Chem 3D Pro 12.0 software (PerkinElmer Inc., Akron, OH, USA). The three dimensions measured were based on the model axes and the mapped cuboid volume of the molecule was calculated from the results.

### 2.4. Drug Release

50 mg PLA/Amox disks containing 1.23 wt% drug were immersed into 20 mL water or 0.9 % saline solution for the drug release test. The test was carried out for one week. Samples were taken intermittently and the concentration of Amox in water or the saline solution was determined by UV-Vis spectrophotometry under the conditions mentioned before. Three parallel measurements were carried out for each medium and composition.

### 2.5. Microbiology

A 1 McFarland standard concentration bacterial suspension of two bacterial strains (*Streptococcus mutans,* ATCC 25175 and *Aggregatibacter actinomycetemcomitans,* DSM 11122) was made separately with saline solution (in suspension, it is equivalent to approximately 3 × 10^8^ colony-forming units/mL). The suspension was spread onto Schaedler agar plates (Schaedler agar + 5% sheep blood (Biomérieux SA, Craponne, France), upon which the compressed disks were placed subsequently. After 24 h of incubation in anaerobic conditions, the diameter of the inhibition zones was measured. Subsequently, the disks were put onto a new agar plate, also inoculated with 1 McFarland standard concentration bacterial suspension freshly made from each of the above-mentioned bacterial strains. The plates were then put into an anaerobic chamber for 24 h. The procedure of replacement onto a new agar plate was repeated until no inhibition zone could be detected (Figure S1). Three parallel measurements were done for each formulation.

## 3. Results and Discussion

The results are presented in several sections. Physical–chemical factors and processes related to fiber spinning, structure, solubility and distribution of the drug are discussed in the first few sections. Wetting and the penetration of the dissolution medium connected with drug release is considered next, followed by drug release and the microbiological effect in the last section of the paper.

### 3.1. Electrospinning, Composition

The electrospinning of biopolymers containing Amox has been done from various solvents and solvent mixtures. Acetone is frequently used [17,20,21,27], and one rightly assumes that the total amount of solvent evaporates during spinning in this case. However, often solvent mixtures are applied, PCL fibers were spun from the mixture of chloroform and methanol [25], while the combination of THF and DMF [22], as well as dichloromethane and DMF [26] were also used for spinning. We used the combination of DCM and DMSO in this study. However, solvents like DMF and DMSO, added to the mixture to facilitate spinning and the dissolution of the drug, have high boiling points and low vapor pressure. Consequently, they do not necessarily evaporate during the spinning process, thus resulting in a material with a composition different from the intended one.

In order to demonstrate the change of composition during sample preparation, the composition of the material in the various steps of the technology is presented in Table 1 for the selected Amox content. Spinning was done from a mixture of DCM, DMSO, and PLA containing 1.23 wt% Amox, calculated for the amount of PLA. The concentration of Amox in the spinning solution was relatively low, and the solution was clear and homogeneous. DCM evaporated during spinning and a three-component material was left behind containing the PLA fibers, DMSO, and Amox. Here the mutual solubility of the components comes into play, DMSO and PLA are not completely miscible, which makes possible the formation of the fibers, but leaves behind a two-phase material consisting of a PLA/DMSO/Amox and a DMSO/Amox phase. The presence of the latter was shown by the exudation of the solvent during the compression of the fibers into disks if DMSO was not evaporated before, and by various characterization techniques such as DSC and Fourier-transform infrared spectroscopy. Accordingly, some part of the active component, i.e., Amox, was not located within the fibers, but among them, in the DMSO solvent. In the final step of fiber production, DMSO was evaporated in an air-circulating oven and Amox precipitated among the fibers in the form of crystals. Accordingly, the form and location of the active component must be crucial for drug delivery, and it depends on a number of factors, including initial composition, technology, and solubility, etc.

### 3.2. Structure

**Figure 1:**
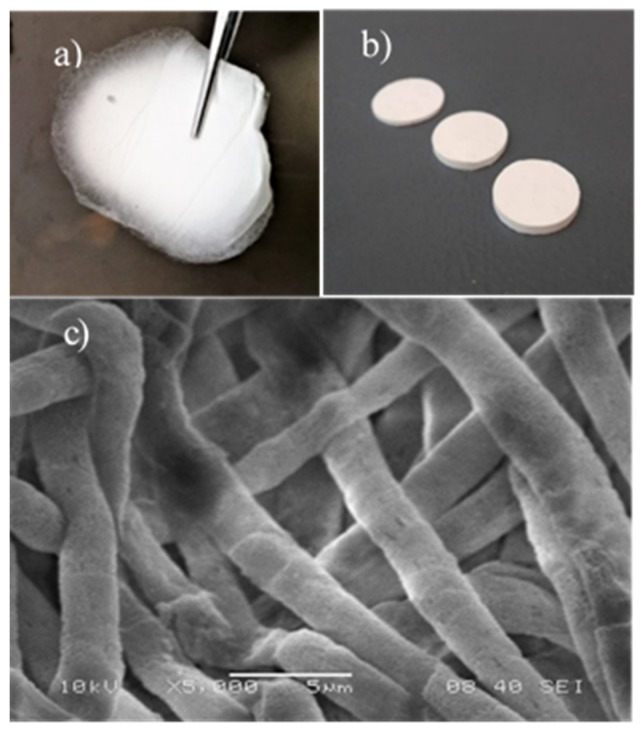
Morphology of PLA/Amox fibers at the different steps of preparation; (**a**) image of the fiber mat; (**b**) image of compressed disks; (**c**) SEM micrograph recorded on the fractured surface of a disk prepared from the electrospun PLA fibers.

The product of the spinning process is a fiber mat (Figure 1a) consisting of an irregular collection of fibers. This is compressed into a disk (Figure 1b) to prepare a device, which is then used for therapy. The drug is crystalline, but the polymer can also crystallize, thus influencing the distribution of the active component and drug release. As Figure 1c shows, the disk prepared consists of closely packed fibers, with some space between, allowing the penetration of the dissolving medium, as well as the diffusion of the drug into the surrounding solution.

**Figure 2:**
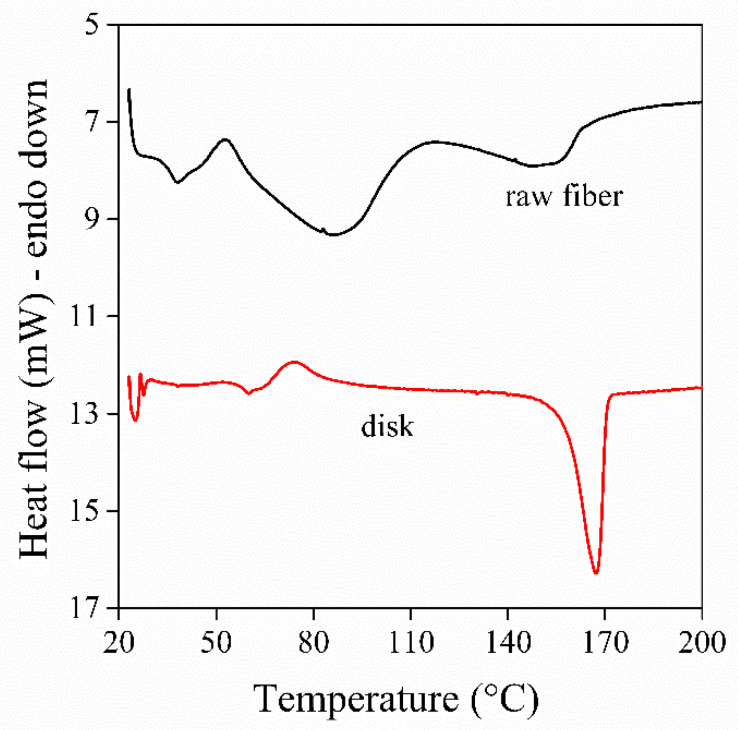
DSC traces recorded on the raw fibers and on a disk prepared from them after the evaporation of DMSO. Amox content: 0.96%.

PLA crystallizes slowly, thus, most products prepared from it usually have an amorphous structure. The fast evaporation of the solvent may also prevent crystallization. The drug is excluded from the crystals, thus, it can be located only in the amorphous phase of the polymer, which also influences release. The DSC traces of the raw fibers and that of the disk prepared from them are presented in Figure 2. The interpretation of the melting curve of the raw fiber is not easy. Several processes can be identified, the glass transition temperature of the polymer, crystallization, evaporation of DMSO and melting. None of the transitions are very sharp and clear because of the presence of a relatively large amount of solvent. The cold crystallization of the polymer indicates that the fibers are mostly amorphous originally. The melting trace of the PLA forming the disk is much simpler and easier to interpret. The glass transition of PLA is followed by a small extent of cold crystallization and then melting at around 165 °C. The melting curve of the disk clearly shows that the crystallinity of the polymer is considerable, it is around 35% compared to that of the raw fiber that is approximately 17%. The change of crystallinity during fiber production must also modify the solubility of the drug in the polymer and thus influence release. 

**Figure 3:**
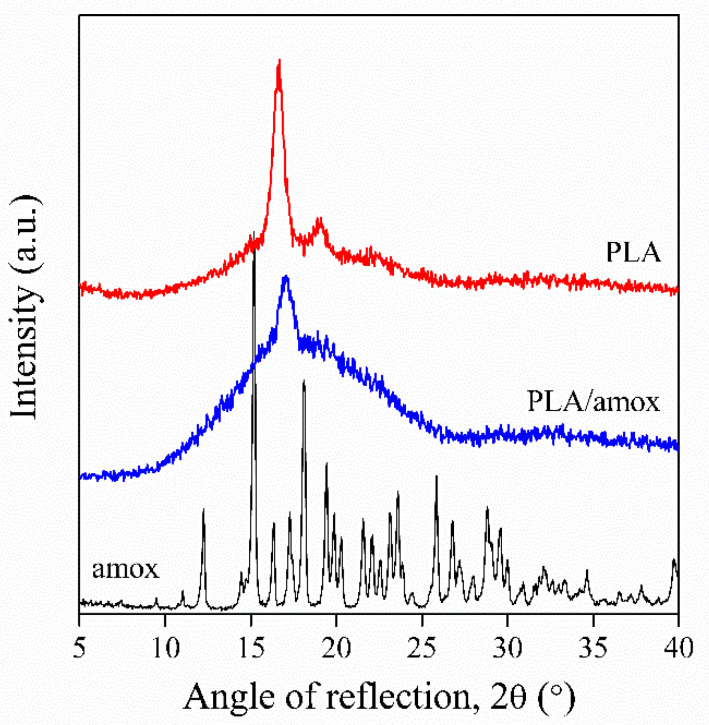
XRD patterns of Amox powder, PLA, and PLA containing 1.23% Amox.

The structure of the components was also checked by XRD. The patterns of the drug, the neat polymer, and that of PLA containing 1.23% Amox are compared in Figure 3. The drug is highly crystalline, which decreases solubility both in the polymer and during therapy. The trace of PLA shows that the disk is crystalline—indeed, the characteristic reflections of the polymer are clearly seen in the XRD pattern together with the usual amorphous halo. The crystallinity of PLA also appears on the pattern of PLA containing the drug, but it seems to decrease somewhat in the presence of the drug that is difficult to explain. The amount of the drug is small and its solubility in PLA is very limited (see later), thus, it cannot greatly influence crystallization, but might interfere with the spinning process and the evaporation of DMSO during drying. The crystalline structure of the components must be considered during the evaluation of the results of drug release experiments.

### 3.3. Solubility

**Scheme 1:**
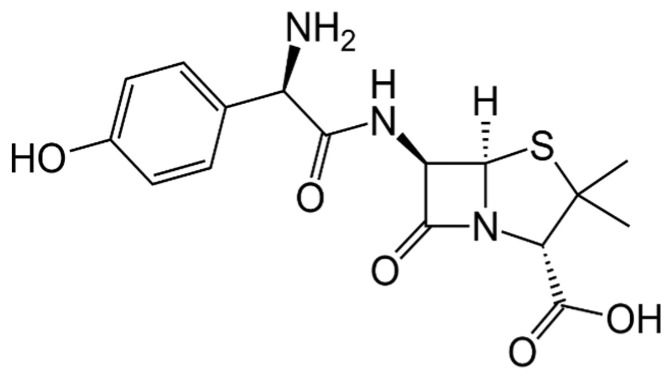
Chemical structure of the amoxicillin molecule.

Small molecular weight drugs are often very polar crystalline substances. The same applies to Amox used in this study, as shown by the structure of the molecule presented in Scheme 1, and by its highly regular crystalline structure indicated by the XRD pattern shown in Figure 3. Polymers, on the other hand, contain far fewer polar groups, thus, their interaction with the drug is usually weak, and the solubility of the drug in the polymer is limited as a result. However, the spinning solution also contains further components, mostly the solvent or solvents, thus, mutual solubility of all components must be considered that is rarely done in studies related to the drug release of scaffolds or wound dressings prepared by electrospinning. 

The interaction of the components can be estimated by various means. One of the simplest approaches is the measurement or calculation of the Hildebrand solubility parameter. The solubility parameter of the components is collected in Table 2. The parameter of most components was calculated with the help of group contributions using the approach of Fedors [29], while that of water was taken from the literature [30]. The solubility parameter for mixtures was calculated from their composition and they are volume average values. According to Table 2, water is the most polar substance involved in the process, but the polarity of Amox and DMSO is also considerable. The solubility parameter of DCM and PLA, on the other hand, is the smallest, and they are very similar to each other, which explains the good solubility of PLA in the solvent, but predicts very limited solubility of the drug in both components. Since the spinning solution is completely homogeneous because of the presence of DMSO, the removal of this latter results in phase separation, the precipitation of the drug.

Solubility was also measured experimentally, wherever it was possible. The measured solubility values are collected in Column 3 of Table 2. The solubility of Amox was the largest in DMSO and still acceptable in the spinning solution, but it was very small in both DCM and the polymer. Accordingly, the drug can exist in the polymer only as a separate phase, in the form of crystals, which of course, strongly influences its release. Solubility in the PLA/DMSO mixture could not be determined experimentally. The relatively small solubility of the drug in water does not affect dissolution and drug release significantly, since the volume of the dissolution medium, as well as in vitro conditions, usually results in very low concentrations.

**Figure 4:**
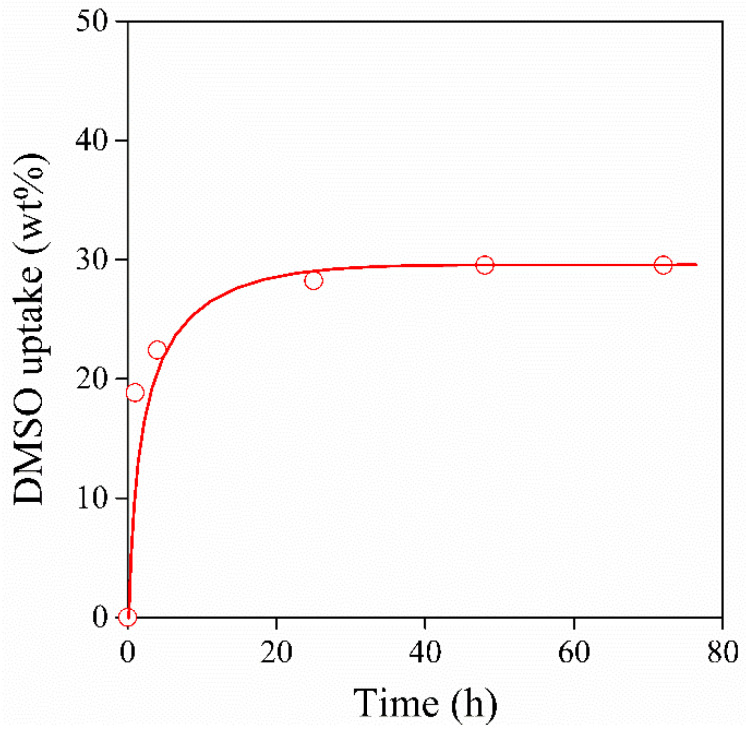
DMSO uptake of a PLA plate plotted as a function of time.

Another factor that must be considered in our specific case is the interaction and mutual solubility of DMSO and PLA. As Table 2 shows, the solubility parameters of the two components differ considerably from each other, thus, DMSO does not dissolve the polymer completely, their miscibility is limited. This statement is strongly supported by Figure 4, showing the DMSO uptake of the polymer as a function of time. The maximum amount of DMSO, which can be dissolved in the polymer is 27.7 wt%, but as Table 1 shows, the raw fibers contain around 64% DMSO. The limited mutual solubility of the polymer and DMSO justifies the phase separation mentioned earlier (see Section 3.1). However, the larger solubility of Amox in DMSO (Table 2) means that a considerable part of the drug is located in the DMSO phase, and it remains among the fibers as precipitated particles upon the evaporation of the solvent. Accordingly, the location and form of the drug at the end of the process producing the disks is crucial for drug release and efficiency.

### 3.4. Location of the Drug

**Figure 5:**
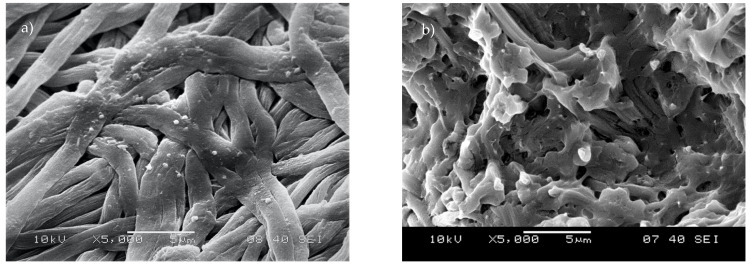
SEM micrographs showing the presence of Amox particles in PLA disks. (**a**) particles located among the fibers, (**b**) particles found within the fibers.

In accordance with the considerations above, the amount of Amox located in the fibers was estimated by taking into account the solubility of the components. After spinning, some DMSO can be found in the fibers, but also as a separate phase. As the solubility of Amox is much larger in DMSO than in the polymer, we must assume that the drug is partitioned in accordance with the amount of DMSO in the phases. These considerations allow the calculation of the theoretical amount of Amox in the fibers, which is listed in the second column of Table 3. The amount of Amox actually encapsulated within the fibers was also determined experimentally. The disks were immersed into excess water in order to dissolve the drug found among the fibers, the fibers were dissolved in DCM, the solution was extracted by water, and then the amount of Amox was determined by UV-Vis spectroscopy. The results are collected in the third column of Table 3. The magnitude of the theoretical and experimental values agree quite well, but the differences, as well as the dependence on Amox content, indicate that factors other than solubility also influence the amount of drug encapsulated in the fibers. These factors must depend on the spinning technology and relate to solvent evaporation, as well as the precipitation and crystallization of the components. The precipitation of the drug and the existence of particles located both among and within the fibers is demonstrated well by the SEM micrographs presented in Figure 5. A large number of small particles can be clearly identified on the surface of the fibers in Figure 5a. However, particles can also be detected within the fibers, as shown by the micrograph on Figure 5b recorded on the cross section of the fibers. Because of the very small solubility of Amox in PLA, the drug can exist only as crystalline particles within the fibers.

The dissolution of the drug from among the fibers and the determination of the amount within the fibers allows the estimation of drug encapsulation efficiency. This latter means the total amount of drug accommodated by the disk prepared from the PLA fibers in this case. The dissolved amount and the encapsulation efficiency are listed in Columns 4 and 5 in Table 3. Similarly to the amount of encapsulated Amox, also the amount of the released drug decreases with increasing concentration of the drug in the spinning solution leading to decreasing encapsulation efficiency. This result means increasing loss with increasing Amox concentration. One source of this is definitely the physical loss of crystalline Amox particles, which was unambiguously observed during the handling of the mats and disks.

### 3.5. Wetting, Penetration

**Figure 6:**
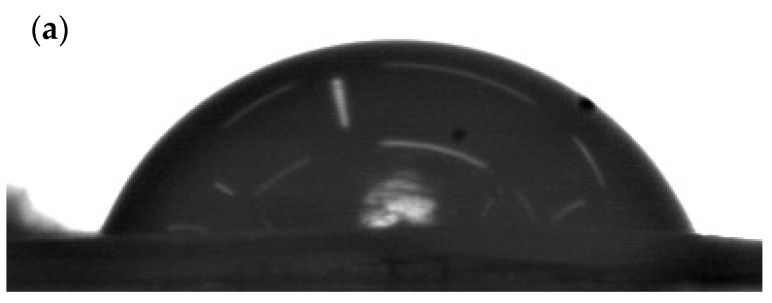

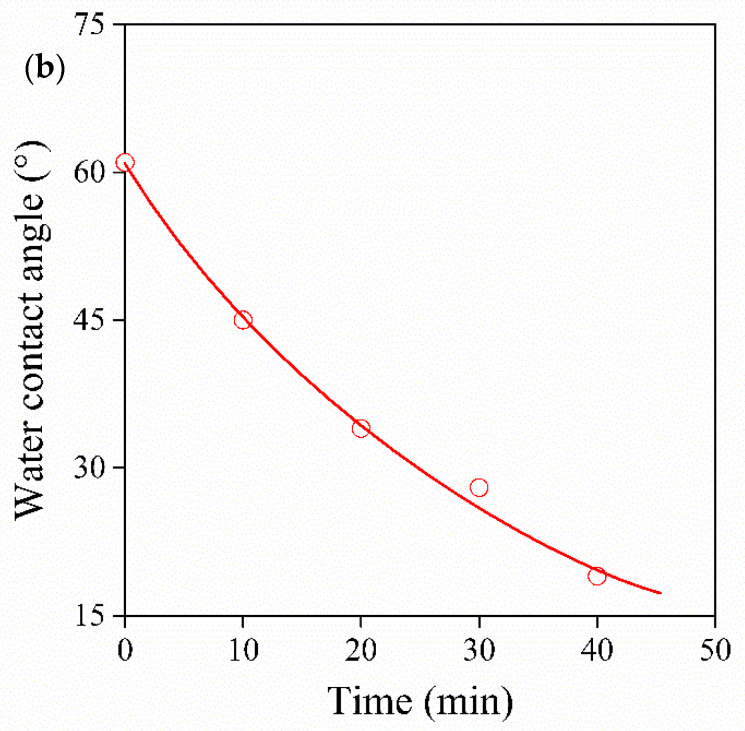
Wetting of a PLA disk prepared from electrospun fibers by water; (**a**) photo of a droplet used for the determination of contact angle, (**b**) decrease of contact angle with time due to the diffusion of liquid into the disk.

The goal of the preparation of the disks is to use them in therapy, for the controlled release of the drug. In order to convey the drug to the location of its action, the surrounding medium, usually body fluid with high water content, must penetrate the disk, dissolve the drug and then this latter must diffuse out of the disk. Because of the crystallinity of the drug, dissolution is not instantaneous and penetration also requires time. PLA is an apolar polymer with a surface energy of 35.5 mJ/m^2^ [31], while water is polar with the surface tension of 72 mJ/m^2^. Accordingly, water does not wet the polymer, as shown by the definite contact angle in Figure 6a. The contact angle is usually measured by placing a droplet of a liquid onto a smooth, stable surface, but these conditions do not apply in our case. The surface used for measurement is neither stable nor smooth, and thus, besides the surface-energy contact angle depends also on pore size and capillary forces. Because of the high surface tension of water, this latter hinders penetration, which may take considerable time. This is demonstrated well by the time dependence of contact angle shown in Figure 6b. The contact angle approached zero, but its determination is very difficult or impossible at longer times, because of its small value and the rough surface of the disk.

**Figure 7:**
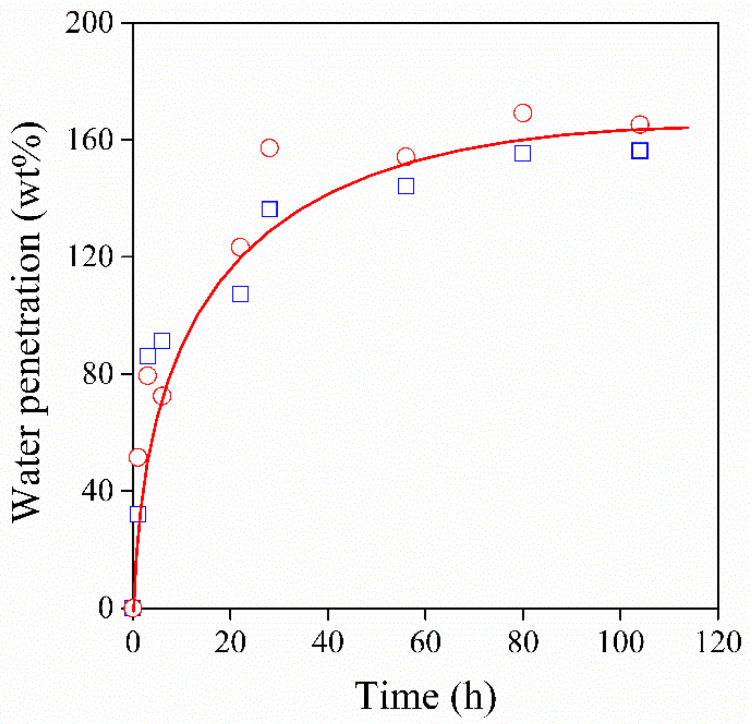
Time dependence of the penetration of water into PLA disks compressed from electrospun fibers. Symbols: (◯) PLA containing 0.96% Amox, (◻) neat PLA.

The time dependence of contact angle clearly shows that the penetration of the medium requires time, but does not allow the determination of complete saturation. Disks were immersed into water and the change of their weight was measured as a function of time. The amount of water taken up by a disk is plotted against the time of the immersion in Figure 7. One can clearly see that saturation is reached in about 120 h and the dissolution of the drug and its diffusion out of the device must last even longer. The permeation of water depends very much also on the preparation conditions of the disks, especially on the compression pressure applied.

### 3.6. Drug Release

**Figure 8:**
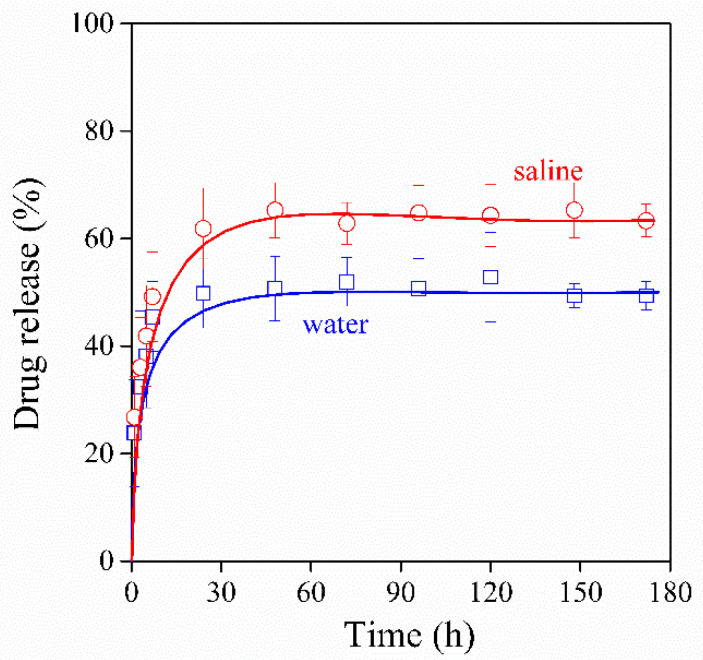
Burst-like drug release from PLA disks prepared from electrospun fibers containing 1.23% Amox.

In most studies, drug release is modeled by immersing fiber mats or devices prepared from them into a dissolution medium, usually water, saline or buffer solution. The release of Amox from the PLA disks prepared in this project is presented in Figure 8 as a function of time. Release increases rapidly initially and then approaches a plateau, indicating maximum release, which is around 50–60% in this case. The plateau is reached in approximately two to three days and we did not observe further release, at least in the time scale of the experiment. The partial release of the drug is in accordance with the considerations presented before, i.e., with the encapsulation of the drug into the PLA fibers resulting in the slow or negligible release, but also by its physical loss during the handling of the disks. The extent of release also depends on the dissolution medium—it is somewhat larger into the saline solution than water. Such an effect of the dissolution medium has been observed before—occasionally, a very small amount of Amox was released into all media [20], but especially into PBS [17].

**Figure 9:**
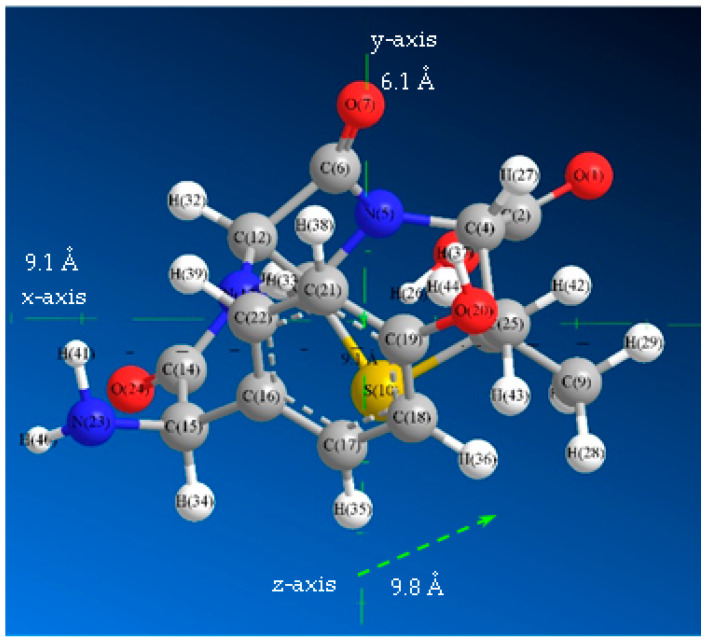
Three-dimensional model of the amoxicillin molecule. Determination of dimensions and the cuboid volume of one molecule.

The fact that a constant value of release is reached needs further consideration. Practically all reports related to the release of Amox from electrospun fibers or related devices claim that release takes place in two steps, a burst-like rapid release and then prolonged-release going on for a long time [17,21,27]. Unfortunately, in the case of PLA/Amox fibers, although a two-stage process is claimed, the experimental evidence does not support it in our case. Amox release reaches a constant value in a couple of days instead of a prolonged release [17,20,26]. The basis of the two-step hypothesis and prolonged drug release is the assumption of slow diffusion of the encapsulated drug from inside the fibers. However, we must thoroughly consider here the possibility and rate of diffusion. The drug molecule presented in Scheme 1 is not small. Its size was estimated by modeling and its dimensions are shown in Figure 9. The volume of the mapped cuboid is 0.544 nm^3^. The molecule must move in the amorphous phase, through the free volume of the polymer. The size of the individual voids creating the free volume was determined as 0.124 nm^3^ using positron annihilation spectroscopy by Kanda et al. [32]. The dissimilarity in the two values indicates very slow diffusion at most, thus, questioning the hypothesis of a two-step release. The situation is different for PCL, since its amorphous phase is above the glass transition temperature allowing the movement of larger molecules as well. However, one may consider the possible occurrence and effect of hydrolysis. The hydrolytic degradation of PLA is slow at neutral pH, but larger or smaller pH as well as other factors, like the catalytic effect of some components, may lead to the degradation of the polymer. As an effect of hydrolysis, the medium usually becomes more acidic, which accelerates degradation further. The degradation of the polymer may result in the slow release of drugs leading to the second slow step observed by many researchers. Unfortunately, composition and conditions are rarely specified, and the possibility of degradation usually is not checked, thus, the reason for the slow release is difficult to identify. Similarly, the method of fabrication may also influence the rate of release, but a detailed study has not been made on this effect either, at least, we are not aware of any such study. Nevertheless, we can clearly state that physical–chemical issues play an important role in the determination of the extent and mode of drug release.

### 3.7. Antimicrobial Activity

**Figure 10:**
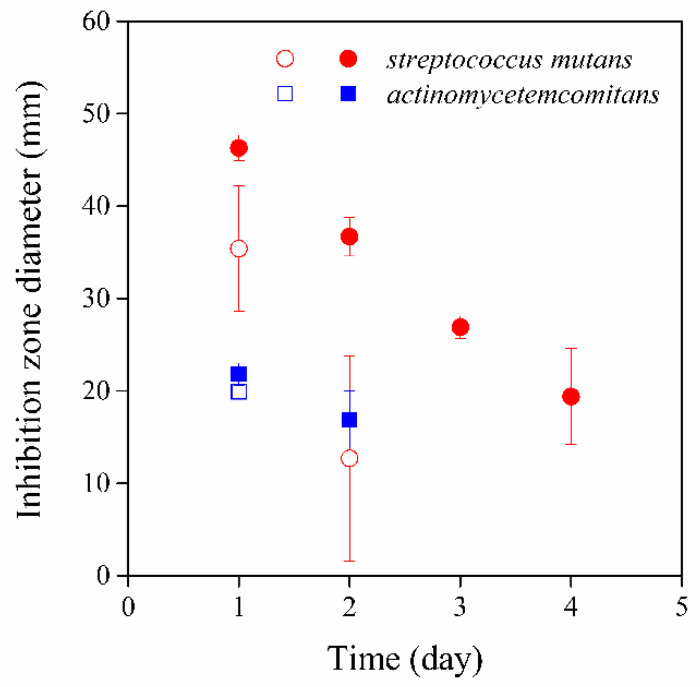
Microbial activity of PLA disks prepared from electrospun fibers with various Amox contents. Symbols: (◯,●) *Streptococcus mutans*, (◻,∎) *Aggregatibacter actinomycetemcomitans*; empty symbols: 0.25 wt%, full symbols: 1.23 wt% amoxicillin.

In order to verify the results presented above, the antimicrobial activity of the disks was determined on two bacterial strains, on *Streptococcus mutans* and *Aggregatibacter actinomycetemcomitans*, as a function of time. The diameter of the inhibition zone was measured after 24 h incubation, and the disks were placed onto a new fresh agar plate daily until any inhibition effect was observed. The results are presented in Figure 10. Amox has different antimicrobial activity towards the two strains, stronger against the *Streptococcus mutans* and somewhat weaker against *Aggregatibacter actinomycetemcomitans*. The results clearly show that the antibacterial activity decreased with time, and higher concentration of amoxicillin in the fibers of the disk led to increased efficiency against *S. mutans.* More importantly, the inhibition effect of the disk with a larger amoxicillin concentration lasted for 4 days in contrast with the two-day effect of the disk with smaller amoxicillin content. Although encapsulation efficiency is lower at higher concentrations (see Table 3), the absolute values in the final device (disk) are larger, and thus the antimicrobial effect is also stronger. No initial dose effect was detected against *A. actinomycetemcomitans*. The time dependence of activity is in close agreement with the results of the drug release experiments showing only short-term effects because of the fast release of Amox located among the fibers of the disk as precipitated crystals.

Based on the microbiological tests, valuable information can also be obtained about the clinical application of the device. By using the PLA/amoxicillin electrospun fibers, a duration of action of 2–4 days can be achieved, which is more favorable than the topically applied conventional preparations (such as the rinses, dental gels), as the latter need to be administered several times a day. Although the release of the active ingredient can be considered to be relatively fast, its duration of action is longer against some microorganisms compared to conventional dosage forms.

## 4. Conclusions

The study focusing on the physical–chemical aspects of drug release from devices prepared by the electrospinning of PLA showed that the composition of the material changes during the preparation procedure. The solubility of the drug, amoxicillin in this case, in the components and that of the components in each other is often limited, which can result in the formation of several phases and the precipitation of the drug either inside or outside the fibers. The composition of the spinning solution consisting of two solvents, DCM and DMSO, results in the partitioning of the active drug, a smaller amount of amoxicillin is precipitated in the form of crystals inside the fibers, while the larger part is located among the fibers. The wetting of the fibers or disks by the water-based dissolution medium is poor, the penetration of the liquid into and the diffusion of the active component out of the device takes considerable time. Drug release takes place in one, burst-like step, practically only the amoxicillin crystals located among the fibers dissolve and diffuse into the surrounding medium. The slow second stage of release claimed in the literature is less probable because the size of the amoxicillin molecule is considerably larger than the holes creating the free volume of the polymer. The prepared device has antimicrobial activity, inhibits the growth of the two bacterial strains studied. The time scale of activity is short and corresponds to that shown by the release experiments and to the burst-like behavior of the device. Although the results clearly prove that physical-chemical factors play a determining role in the effect and efficiency of medical devices prepared from electrospun fibers containing an active component, the study shows that the selected combination of materials and techniques have some limitations as well. Probably, a larger effect and efficiency could be achieved if a different polymer or a polymer blend having larger miscibility with Amox were selected as carrier material.

## Figures and Tables

**Table 1:** Composition of the material at the different stages of the preparation process.

Material	Component (wt%)			
	DCM	DMSO	PLA	Amox
Spinning solution	75.49	15.61	8.80	0.11
Raw fiber		63.66	35.89	0.45
Dried fiber			98.77	1.23

**Table 2:** Interactions and solubility of Amox in the various components.

Component	Solubility Parameter, δ (MPa)^1/2^	Solubility, c_s_ (wt%)
Water	47.9	0.36
Amoxicillin	36.7	n.a.
DMSO	26.7	3.70
PLA/DMSO	24.5	–
Spinning solution	21.4	0.22
DCM	20.2	0.02
PLA	20.2	0.01

**Table 3:** Location of amoxicillin in electrospun PLA fiber disks; encapsulation, release, efficiency.

Amox Content (wt%)	Within PLA (%)		Released (%)	Encapsulation (%)
	Theoretical	Extracted		
0.25	15.5	22.5	72.5	95.0
0.50	15.5	22.2	65.7	88.0
0.96	15.6	14.2	53.2	67.4
1.23	15.7	11.1	48.8	59.9

## Data Availability

Not applicable.

## Supplementary Materials

The following are available online at https://www.mdpi.com/article/10.3390/pharmaceutics13101645/s1, Figure S1: Schematic graph of the microbiological study.
