# Supplementary material for: Physical–Chemical Aspects of the Preparation and Drug Release of Electrospun Scaffolds
Source: Pharmaceutics. 2021 Oct 9;13(10):1645. doi: 10.3390/pharmaceutics13101645 (PMC8540048; doi:10.3390/pharmaceutics13101645)
Supplement: Supplementary file 1 [file pharmaceutics-13-01645-s001.zip › pharmaceutics-1378478-supplementary.pdf]

pharmaceutics

Supplementary Materials: Physical-Chemical Aspects of the Preparation and Drug Release of Electrospun Scaffolds

Lu Cui, Judit Rebeka Molnár, Mária Budai-Szűcs, Mária Szécsényi, Katalin Burián, Péter Vályi, Szilvia Berkó and Béla Pukánszky

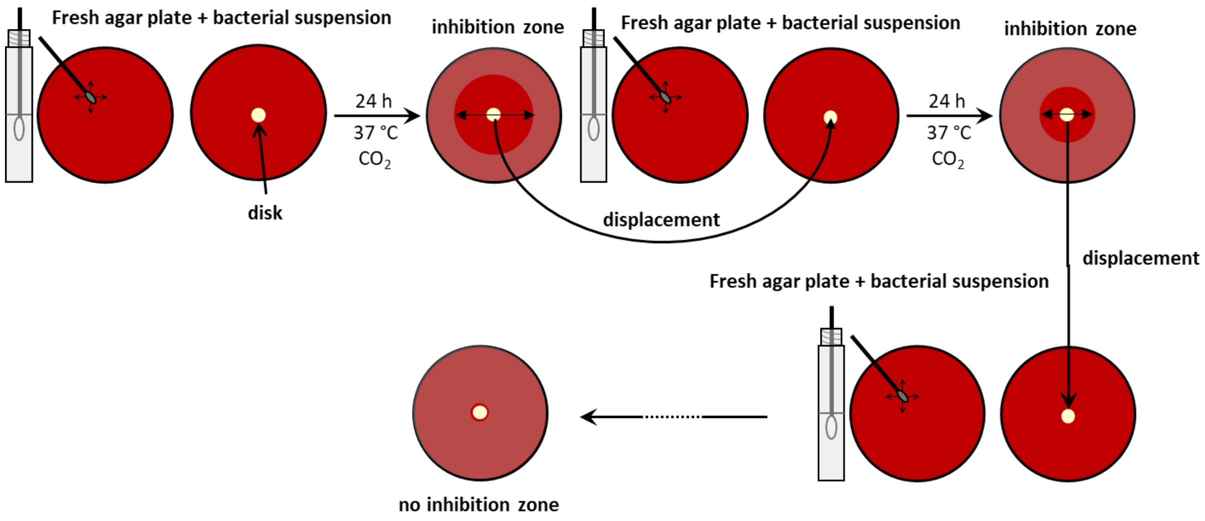

Figure S1. Schematic graph of the microbiological study.
